# MLL rearrangements in pediatric acute lymphoblastic and myeloblastic leukemias: MLL specific and lineage specific signatures

**DOI:** 10.1186/1755-8794-2-36

**Published:** 2009-06-23

**Authors:** Andrea Zangrando, Marta Campo Dell'Orto, Geertruy te Kronnie, Giuseppe Basso

**Affiliations:** 1 Laboratory of HematoOncology, Department of Pediatrics "Salus Pueri", University of Padova, Padova, Italy

## Abstract

**Background:**

The presence of *MLL *rearrangements in acute leukemia results in a complex number of biological modifications that still remain largely unexplained. Armstrong et al. proposed *MLL *rearrangement positive ALL as a distinct subgroup, separated from acute lymphoblastic (ALL) and myeloblastic leukemia (AML), with a specific gene expression profile. Here we show that MLL, from both ALL and AML origin, share a signature identified by a small set of genes suggesting a common genetic disregulation that could be at the basis of mixed lineage leukemia in both phenotypes.

**Methods:**

Using Affymetrix^® ^HG-U133 Plus 2.0 platform, gene expression data from 140 (training set) + 78 (test set) ALL and AML patients with (24+13) and without (116+65) *MLL *rearrangements have been investigated performing class comparison (SAM) and class prediction (PAM) analyses.

**Results:**

We identified a *MLL *translocation-specific (379 probes) signature and a phenotype-specific (622 probes) signature which have been tested using unsupervised methods. A final subset of 14 genes grants the characterization of acute leukemia patients with and without *MLL *rearrangements.

**Conclusion:**

Our study demonstrated that a small subset of genes identifies *MLL*-specific rearrangements and clearly separates acute leukemia samples according to lineage origin. The subset included well-known genes and newly discovered markers that identified ALL and AML subgroups, with and without *MLL *rearrangements.

## Background

The *MLL *gene located on chromosome 11 band q23 normally functions as a transcription regulator of the HOX genes [1] and is essential for normal mammalian development and hematopoiesis [2]. Chromosomal translocations involving *MLL *gene represent frequent cytogenetic abnormalities found in hematologic malignancies, occurring in 5–6% of patients with acute myeloid leukemia (AML), 7–10% of acute lymphoblastic leukemia (ALL), 60–70% of all acute leukemias in infants, and in most patients with t-AML/t-ALL secondary to therapy that is targeting topoisomerase II [3]. The function of the various *MLL *fusion genes [4,5] and proteins is poorly understood but it appears that the fusion proteins disrupt the ability of wild-type *MLL *to regulate HOX gene expression, leading to leukemogenesis [6]. Recent studies demonstrated that the presence of *MLL *rearrangements can be associated to specific antigen [7,8] and gene expression patterns. Pediatric patients with ALL carrying *MLL *rearrangement have been successfully distinguished from ALL and AML patients without *MLL *translocation as a distinct subgroup with a specific gene expression profile [9]. Different gene expression signatures for ALL and AML samples, with and without *MLL *translocation were also identified in adult patients indicating a common method to comprehensively characterize the *MLL *mutation [10]. However, the involvement of *MLL *gene in the onset and progression of leukemia event still remains unclear. The present study encompasses the efforts to clarify the relations between *MLL *translocation and acute leukemias in pediatric patients: we identified common MLL-related markers that are shared between leukemias with different phenotypes (translocation-related signature) and investigated the role of *MLL *aberration in acute leukemias with different lineage origin (phenotype-related signature). To this end, two independent cohorts of 140 (training set) and 78 (test set) pediatric patients with ALL/MLL+, AML/MLL+, ALL/MLL- and AML/MLL- have been inspected using gene expression profiling. Separated comparisons based on phenotype and translocation information have been applied to find differentially expressed genes using both comparison (SAM) and prediction (PAM) analyses. Each subgroup has been clearly distinguished using a final subset of 15 probes which separated the training cohort samples into phenotype-related and translocation-related signatures. The strength of our predictor was successfully validated on an independent test cohort. The identified markers have been further examined to explain their biological correlations using gene ontology inquiries. We assessed the key role of previously unexplored genes to specifically characterize *MLL *translocation as well as the impact of well-known genes in separating acute leukemia samples according to phenotype origin.

## Methods

### Patients and Samples

A total cohort of 140 pediatric patients was enrolled in the training set. Bone marrow samples were collected at diagnosis from 106 and 34 patients with acute lymphoblastic and myeloblastic leukemia, respectively. The presence of *MLL *rearrangement was detected in 16/116 (13.8%) ALL patients and in 8/34 (23.5%) AML cases (Table 1). Detailed sample information are provided in Additional File 1. A second cohort of 78 patients has been used as test set to validate the results from supervised analyses performed on the training set.

**Table 1:** Patient cohort

	**N° patients**	**Gender**	**Median Age**
**Training cohort**	140	70 M/70 F	6.2 y (0.1 – 17)

**ALL/MLL-**	90	48 M/42 F	6.51 y (0.88 – 16.5)

**AML/MLL-**	26	12 M/14 F	8.42 y (0.45 – 17)

**ALL/MLL+**	16	8 M/8 F	2.14 y (0.1 – 14.5)

**AML/MLL+**	8	2 M/6 F	3.59 y (0.16 – 13.6)

Patient distribution of the training cohort according to phenotype and *MLL *translocation.

For all patients, ALL and AML diagnoses were performed by morphology, cytochemistry, cytogenetics, immunophenotype and molecular genetics.

Based on the laboratory diagnosis, patients were risk stratified and enrolled in the following AIEOP protocols: LAL2000 (ALL after 2000 year), LAM2002 (AML after 2002 year), LAL95 (ALL before 2000 year), LAM92 (AML before 2002 year) and Interfant99 (ALL and AML less than 1 year old) [11-13]. This study was conducted after obtaining the informed consent from all patients and following the tenets of the Declaration of Helsinki and was approved by the ethics committees of the participating institutions before the initiation.

### Morphological classification

The morphological classification was performed by three independent investigators and the conclusive diagnosis for every case was reported according to the FAB criteria. In cases where consensus was not obtained, the three investigators re-analyzed the slides together in order to obtain consensus as to final diagnosis.

### Cytogenetic analyses

Samples were processed and cytogenetic studies were performed using the Q-banding technique. About 15–20 metaphases for each sample were acquired (with CASTI System) and analyzed in order to avoid clone loss. Chromosomes were identified and assigned according to the International System for Human Cytogenetic Nomenclature. FISH analysis was performed on interphase nuclei and where possible on metaphases, using an *MLL *probe (Vysis).

### Biomolecular analyses

The AIEOP MLL protocol, which screens for the fusion gene transcripts *MLL-AF4 *for t(4;11) translocation, *MLL-AF10 *for t(10;11), *MLL-AF9 *for t(9;11) and *MLL-ENL *for t(11;19), was performed as stated in the previously reported method; briefly, total RNA was isolated using the RNAzol-B reagent (Duotech srl Milan, Italy) following manufacturer's instructions. One microgram of total RNA from each specimen was reverse transcribed using the Superscript reverse transcriptase (Life Technologies Milan, Italy) and random hexamers. PCR amplification was performed using AmpliTaq polymerase (Applied Biosystems) according to the BIOMED-1 protocols. An independent PCR reaction was performed with shift primers for confirmation of each positive result. The ABL housekeeping gene expression was assessed to determine the presence of amplifiable RNA and the efficacy of reverse transcriptions. After electrophoresis, the PCR reaction products were stained with ethidium bromide.

### RNA isolation for microarray analysis

Microarray analysis was performed on each sample using Affymetrix Human Genome U133 Plus 2.0 GeneChip. Total RNA was extracted from stored frozen cells of leukaemia specimens using TRIzol (Invitrogen) followed by a purification step (RNeasy Mini Kit, Qiagen). RNA quality was assessed on the Agilent Bioanalyzer 2100 using the Agilent RNA 6000 Nano Assay kit (Agilent Technologies, Waldbronn, Germany). RNA concentration was determined using the NanoDrop ND-1000 spectrophotometer (NanoDrop Technologies, Inc., Wilmington, DE USA). The overall total RNA quality was assessed by A260/A280 ratio.

### Study aim and design

**Figure 1:**
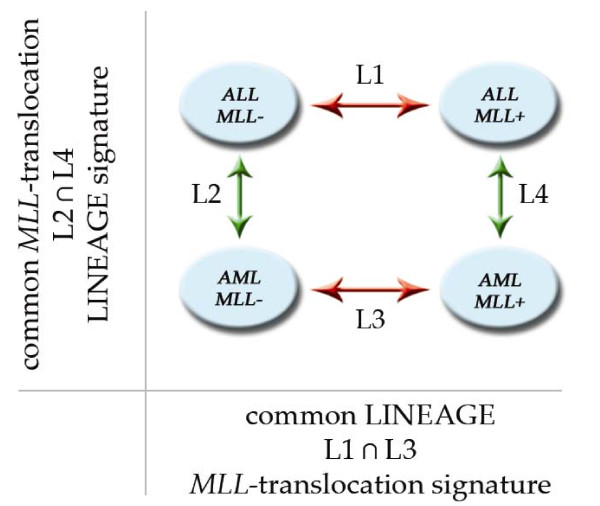
**Study aim and design**. Study aim and design for supervised analyses. MLL signature was obtained by comparing samples with the same phenotype (red arrows) while lineage signature by comparing samples with and without *MLL *translocation (green arrows).

We performed comparisons between subgroups carrying the same lineage (Figure 1, ALL/MLL- vs ALL/MLL+ named "L1"; AML/MLL- vs AML/MLL+ named "L3") or the same translocation information (ALL/MLL- vs AML/MLL- named "L2"; ALL/MLL+ vs AML/MLL+ named "L4") using SAM and PAM algorithms. Combining the results from separated lineage-related comparisons (L1 and L3), we identified a specific MLL signature that is shared between ALL and AML subtypes. Matching the gene lists from translocation-specific comparisons (L2 and L4), we described a common lineage signature for samples with and without *MLL *translocation.

The results from SAM and PAM analyses have been tested on an independent cohort to separate samples according to their gene expression profiles.

### Gene Expression Profiling

Microarrays analyses were performed using Human Genome U133 Plus 2.0 GeneChip Array (Affymetrix, Santa Clara, CA, USA) and specific RNA isolation method as previously described. Microarray data (CEL files) can be found online at GEO repository (Accession Number: GSE14062).

Statistical analyses were performed using open-source BioConductor package [, ver.2.1] for R System software [The R Project for Statistical Computing, ver.2.7.0, ].

The training and test sets were inspected using the same analysis procedures. Microarray CEL files were analyzed to evaluate potential errors on the arrays using quality control algorithms on PS intensity signals. To allow gene expression comparison, the robust multiple-array average (RMA) normalization has been performed among all arrays. Genes with low expression variation across all samples have been discarded using interquartile range (IQR>1.15) as filtering criteria; the following analyses have been performed starting from 5130 filtered probes.

Significance analysis of microarray (SAM) package has been used in the training set to find differentially expressed genes among ALL/MLL-, AML/MLL-, ALL/MLL+ and AML/MLL+ groups. Less than one false positive-rated gene was found using false discovery rate (FDR) <1% and q-value = 0 cutoffs.

Obviously genes identified by SAM analysis are not necessarily involved in class prediction [14]. Shrunken centroid algorithm was performed on the training set using Tibshirani's prediction analysis of microarrays (PAM) package. All probes but one (204069_at, one of the two *MEIS1 *probes included in the prediction list) identified by PAM analysis matched the SAM results. The results from supervised analyses were further validated on an independent test set. SAM gene lists were used to perform gene ontology studies using the Database for Annotation, Visualization and Integrated Discovery (DAVID) web-based tool . The phenotype-related signature was analyzed through the use of Ingenuity Pathway Analysis (Ingenuity^® ^System, ).

## Results

### SAM results – Training Set

The training cohort of 140 samples was analyzed using Affymetrix HG-U133 Plus 2.0 gene expression chips. As previously described in the study design, four separated comparisons (labeled L1 to L4) were performed using SAM and PAM methods to identify differentially expressed probes for translocation-specific and lineage-specific groups. Table 2 summarizes the results retrieved by SAM analysis using a false discovery rate (FDR) < 1% and q-value = 0 as statistical cut-off values.

**Table 2:** SAM results

*Constant part*	Phenotype	Phenotype	Translocation	Translocation
*Variable part*	Translocation	Translocation	Phenotype	Phenotype

*Comparison ID*	L1	L3	L2	L4

*SAM comparisons*	ALL/MLL(-) vs ALL/MLL(+)	AML/MLL(-) vs AML/MLL(+)	ALL/MLL(-) vs AML/MLL(-)	ALL/MLL(+) vs AML/MLL(+)

***up***/*down ALL/MLL(-)*	**1013**/740		**1378**/754	

***up***/*down AML/MLL(-)*		**155**/555	**754**/1378	

***up***/*down ALL/MLL(+)*	**740**/1013			**379**/601

***up***/*down AML/MLL(+)*		**555**/155		**601**/379

Total	*1753*	*710*	*2132*	*980*

*(Common) Signature*	**379 **Translocation specific		**622 **Phenotype specific	

SAM results for comparisons between considered subgroups. Translocation-specific signature was obtained by matching deregulated probe sets from L1 and L3 comparisons, phenotype-specific signature from L2 and L4 comparisons.

To identify a *MLL *translocation-specific signature commonly shared by different lineage subtypes, we compared ALL and AML samples separately. For each comparison, upregulated probes in one group are consequently downregulated in the other, and vice versa. The results from ALL/MLL- vs ALL/MLL+ (L1 comparison: 1013 upregulated probes in the former group, 740 upregulated in the latter) and AML/MLL- vs AML/MLL+ (L3 comparison: 155 overexpressed in the first group, 555 overexpressed in the second) were subsequently matched to generate a unique MLL-specific subset. A total of 379 common translocation-specific probe sets were found from L1 and L3 comparison combining 1753 and 710 probes, respectively [see Additional File 2].

The phenotype-associated signature was identified by comparing samples with and without *MLL*-mutations separately. We matched the results from ALL/MLL- vs AML/MLL- (L2 comparison: 1378 upregulated probes in the former group, 754 upregulated in the latter) and ALL/MLL+ vs AML/MLL+ (L4 comparison: 379 overexpressed in the first group, 601 overexpressed in the second), obtaining 622 shared phenotype-specific probe sets from 2132 (L2) and 980 (L4) differentially expressed markers [see Additional File 2].

**Figure 2:**
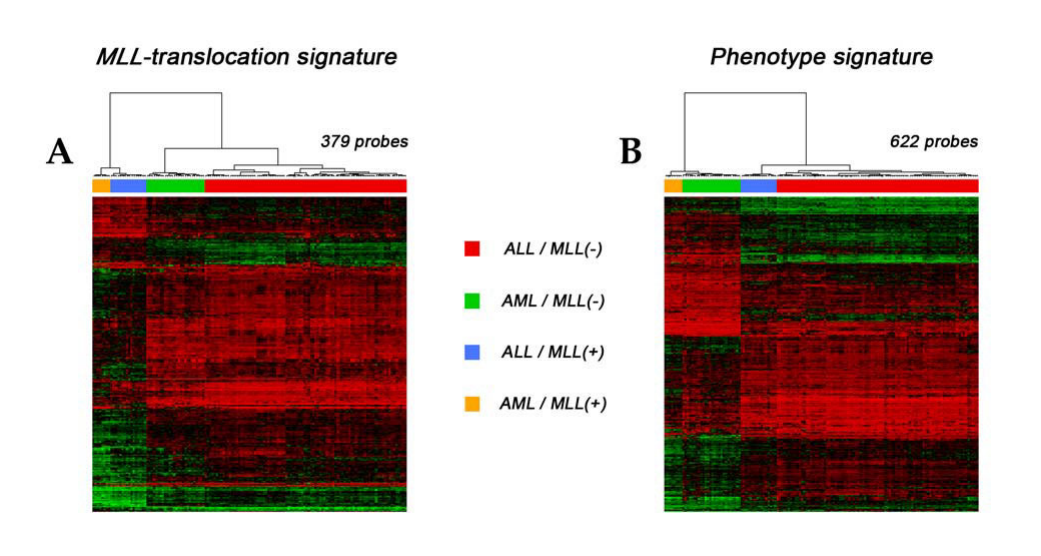
**MLL-specific and Phenotype-specific signatures**. Unsupervised hierarchical clustering performed on 140 samples using (**A**) translocation-specific (379 probe sets) and (**B**) phenotype-specific (622 probe sets) signatures. Cluster A clearly separates patients with *MLL *(orange and blue labels) from patients without *MLL *translocation (green and red labels). Cluster B distinguishes samples with AML (orange and green labels) from samples with ALL (blue and red labels).

SAM results were tested on 140 samples using an unsupervised hierarchical clustering method: patients were clearly distinguished in two groups according to translocation- (379 probes, Figure 2A) and phenotype-related (622 probes, Figure 2B) signatures.

### PAM results – Training Set

SAM comparisons were reproduced in the training cohort using a prediction algorithm (PAM). We identified a total of 15 probe sets, corresponding to 14 genes, with predictive value in separating ALL and AML with and without *MLL *rearrangement (Table 3). Fourteen out of 15 probes extracted by PAM matched SAM analyses results [see Additional File 2].

**Table 3:** PAM results

**PAM comparisons**	ALL/MLL- vs ALL/MLL+	ALL/MLL- vs AML/MLL-	AML/MLL- vs AML/MLL+	ALL/MLL+ vs AML/MLL+
**Prediction ID**	L1	L2	L3	L4

**Class Error Rate**	0%	0%	0%	0%

**Probes identified**	2	2	7	4

**Gene symbol**	*MEIS1, MEIS1*	*TCL1A, EBF1*	*ZEB2, SRGAP2P1, AK2, TMEM30A, FAM62B, TMED2, HIPK3*	*PAX5, CD72, LOC100130458, CSRP2*

Class prediction analyses (PAM) performed on the training cohort of 140 samples using 5130 filtered probes.

**Figure 3:**
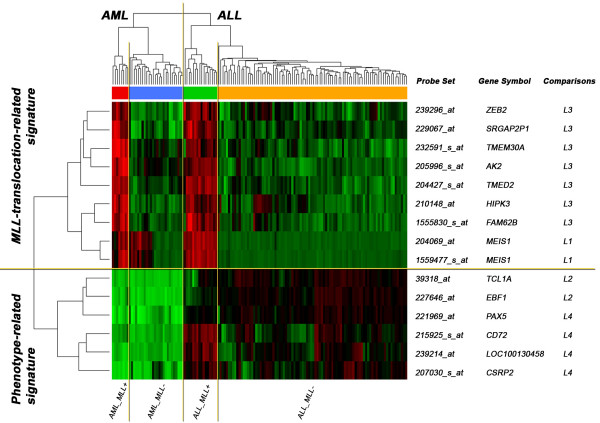
**Hierarchical clustering for PAM results**. Unsupervised hierarchical clustering using the 15 probe sets identified by PAM analyses on the training set. Each column identifies a patient, each row a probe set. The upper dendrogram separates AML (red and blue labels) from ALL (green and orange) samples. Each group further divides into MLL-positive and MLL-negative samples. The dendrogram on the left groups probe sets according to phenotype- and *MLL *translocation-related signatures.

To test prediction performances, we applied unsupervised hierarchical clustering on 140 samples using PAM results. In Figure 3, the upper dendrogram clearly separated our samples into 2 branches according to ALL (106 samples) and AML (34 samples) phenotypes. Each branch further divided into MLL positive and MLL negative subgroups showing distinct gene expression profiles. Additionally, the probe sets retrieved by PAM analysis were associated into 2 major clusters as shown in the dendrogram on the left. A translocation-related signature was characterized by overexpression of *ZEB2*, *SRGAP2P1*, *TMEM30A*, *AK2*, *TMED2*, *HIPK3*, *FAM62B *and *MEIS1 *genes in MLL positive samples both ALL and AML, while a common upregulation of *PAX5*, *CD72*, *CSRP2*, *LOC100130458*, *TCL1A *and *EBF1 *genes correlated with ALL patients in a phenotype-related signature.

### Validation on Test Set

The predictor (15 probe sets) obtained by SAM and PAM results has been used for validation on a separated cohort of 78 samples. Patients were distributed as follows: ALL/MLL- n = 54, AML/MLL- n = 11, ALL/MLL+ n = 8, AML/MLL+ n = 5. The dendrogram in Additional File 3 validates the strength of our predictor in discriminating ALL and AML with and without *MLL *rearrangements. Only one sample (PD529) has been misclassified in the independent cohort, probably due to the small sample size of AML/MLL+ subgroup.

### MLL Translocation-related signature

ALL patients with and without *MLL *translocation have been compared (L1). Two probes for *MEIS1 *gene (204969_at, 15559477_s_at), which encodes a cofactor for HOX proteins that can accelerate Hoxa9-dependent leukemia, were shown to be strongly expressed in MLL positive samples, as previously reported [15]. *MEIS1 *quantitatively regulates the differentiation arrest, cycling activity, in vivo progression, and self-renewal of MLL leukemia cells, thereby functioning as a critical and rate-limiting determinant of leukemia stem cell potential [16].

*ZEB2 *(SIP1) gene, coding a zinc finger E-box binding homeobox 2 protein, was already reported as cancer activating factor. In synergy with another transcription factor (Snail), *ZEB2 *represses transcription of the E-cad gene by binding E-box on E-cad promoter. Loss of E-cadherin (E-cad) triggers invasion, metastasis, and dedifferentiation in various epithelial carcinomas [17].

Interestingly *AK2 *and *HIPK3 *genes, here upregulated in *MLL *rearrangements, share the same target in Fas-mediated apoptosis pathway, an adaptor molecule (FADD) that interacts with various cell surface receptors and mediates cell apoptotic signals. Adenylate kinase 2 (*AK2*) regulates mitochondrial apoptosis through the formation of an AK2-FADD-caspase-10 (AFAC10) complex. Acting in concert with FADD and caspase-10, *AK2 *mediates a novel intrinsic apoptotic pathway that may be involved in tumorigenesis [18]. Another Fas/FADD-interacting kinase, *HIPK3 *(*PKY*), was first identified as a putative multidrug-resistant protein from studies of cancer cells. Common death receptor target of *AK2 *and *HIPK3 *suggests that a principle role of this kinase family is in regulating various aspects of death receptor signaling [19,20]. The upregulation of *AK2*, *MEIS1 *and *TMEM30A *(transmembrane protein 30A) genes in *MLL*-rearranged acute leukemias has also been demonstrated by Faber et al. [21] using gene expression profiling.

The genes *SRGAP2P1 *(SLIT-ROBO Rho GTPase activating protein 2 pseudogene 1), *TMED2 *(transmembrane emp24 domain trafficking protein 2) and *FAM62B *(family with sequence similarity 62 (C2 domain containing) member B) shared similar upregulation in both ALL and AML with *MLL *translocation but their role in leukemogenesis remains to be explored.

### Expression of HOX and NG2 genes

HOX gene expression was evaluated in our patient cohort: *HOXA10*, *HOXA9*, *HOXA7*, *HOXA5 *and *HOXA3 *were found to be generally upregulated in samples carrying *MLL *translocation, both in ALL and AML, while *HOXA11 *and *HOXA6 *showed common overexpression in AML/MLL+ cases [16]. *NG2 *gene, an integral membrane chondroitin sulfate proteoglycan expressed by human malignant melanoma [22] and leukemic [7,23] cells, exhibited higher expression in both ALL and AML with *MLL *rearrangement similar to HOX genes (data not shown).

### Phenotype-related signature

A total of 6 probe sets with predictive value were isolated using PAM analysis to separate ALL from AML samples with and without the presence of *MLL *aberration. All probes showed a marked downregulation in AML samples as well as a common involvement in enhancing the B-cell signaling pathway.

*PAX5 *plays a key role in regulating a number of genes identified by PAM and SAM analyses distinguishing ALL from AML phenotypes. *PAX5 *gene codes for BSAP, a transcription factor expressed in the developing central nervous system, testis and cells of B lymphocyte lineage except terminally differentiated plasma cells [24,25]. BSAP-binding sites have been identified in several genes, included in SAM list, encoding *VpreB1 *[26], *BLK *[27,28], *CD79a *[29], *CD19 *[30], *EBF *[31], *CD72 *[32], *BLNK *[33,34] and *LEF1 *[35]. CD72 antigen is a transmembrane glycoprotein that plays a fundamental role in B-cell activation and proliferation. It has been recently shown its function also in preventing the differentiation of naïve B-cells into plasma cells [36]. CD19 antigen is routinely monitored in immunophenotyping diagnosis as the main surface marker for B cell identification. The CD19 tyrosine phosphorylation is induced by CD72 ligation: the activation of B lymphocytes via CD72 resulted in recruitment and activation of PI 3-K, which was mediated by CD19. Moreover, CD19 enhances membrane IgM signaling like CD79a even known as Igα [37]. BLNK is a B-cell linker protein which is fundamental in addressing pro-B cell to pre-B cell transition [38]. Kabak et al. [39] demonstrated the direct recruitment of BLNK to Igα (CD79a) in signaling pathways and Imai et al. [40] showed that BLNK expression is a common leukogenic event in childhood B-lineage ALL similarly to the B lineage-specific polymerase encoded by *DNTT *gene.

**Figure 4:**
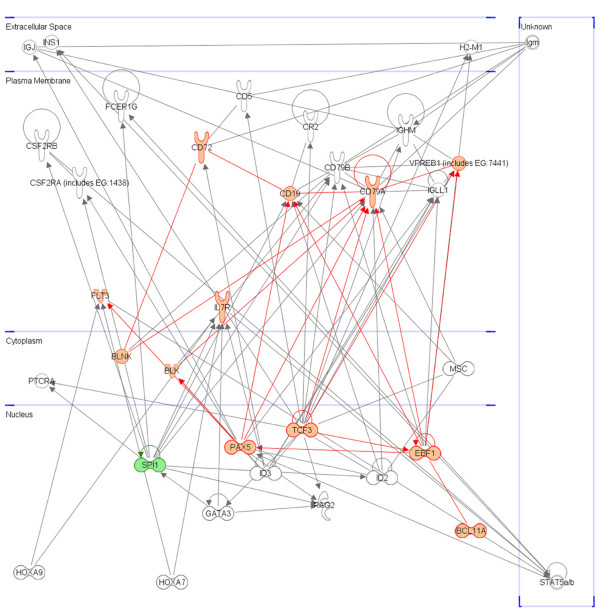
**Genes involved in pro-B cell differentiation and proliferation**. Regulatory role of *PAX5 *in B cell differentiation and proliferation. Up-regulated and down-regulated genes in ALL/MLL+ from supervised analyses are depicted red and green, respectively. The minimal self-sustaining gene regulatory module was obtained by Medina K. et al. [36] and expanded according to our results. The graph was generated using Ingenuity Pathways Analysis (IPA) software (Ingenuity^® ^System, ).

To assess our findings, we compared PAM and SAM results to a minimal self-sustained gene regulatory module for pro-B cell differentiation and proliferation proposed by Medina K. et al. [41]. All module genes (*FLT3*, *PU.1*, *E2A*, *BCL11A*, *IL-7R*, *EBF*, *PAX5*) except *PU.1 *were included in SAM lists showing significant over-expression in MLL+/ALL group (Figure 4).

*CSRP2 *is a member of the CSRP (cysteine and glycine-rich protein) family of genes, encoding a group of LIM domain proteins, which may be involved in regulatory processes important for development and cellular differentiation but also in oncogenesis [42].

During lymphocyte differentiation, the expression of T-cell leukemia/lymphoma 1A gene (*TCL1A*) begins in pre-B cells, is downregulated in germinal centre B cells, and is silenced in memory B and plasma cells [43-45]. Performing comparative Gene-Set Enrichment Analysis (GSEA), Aggarwal et al. [46] demonstrated that the increased expression of *TCL1A *was significantly associated (P < 0.05) with some of the most important pathways controlling B-cell lymphoma pathogenesis and heterogeneity, including B-cell receptor pathway.

### Gene Ontology results

We further inspected the SAM gene lists using a web-based gene ontology (GO) tool (DAVID) to understand the biological meaning behind large list of genes. Additional File 4 summarizes the GO results (Biological Processes) for both SAM signatures ordered by FDR, defined as the median number of false positive genes divided by the number of significant genes.

The analysis of lineage-specific signature (distinguishing ALL from AML) clearly shows the involvement of hematopoietic and immune response-related genes in the progress of disease, in particular leukocyte activation and hematopoietic organ development. MLL-related signature, distinguishing samples with and without *MLL *translocation, mainly includes regulatory genes involved in developmental process, biological process, apoptosis and programmed cell death.

### AML subgroup without MLL translocation and MEIS1 upregulated

In the present study we showed *MEIS1 *gene to be upregulated both in ALL and AML with *MLL *translocation. As depicted in Figure 3, a subgroup of AML without *MLL *mutation displayed clear overexpression for both *MEIS1 *probes (as well as *HOXA9 *and *HOXA5 *genes, see Additional File 5), confirmed by supervised analysis on the 26 patients with AML/MLL-. Patient distribution is described in Table 4: interestingly, all patients with t(8;21)(q22;q22) and t(15;17)(q22;q21) correlated with down-regulation of *MEIS1*, while 3 out of 4 patients with inv(16)(p13q22) highly expressed *MEIS1 *gene. Our results confirm the observations by Grubach et al. [47] who determined RQ-PCR expression levels of a series of PcG genes (including *MEIS1*) and PcG-regulated genes in 126 AML patients and 20 healthy donors.

**Table 4:** AML without MLL and MEIS1 upregulated

**AML without MLL**	***MEIS1*-upregulated group**	***MEIS1*-downregulated group**
*Normal*	8	5

*inv(16)(p13q22)*	3	1

*t(8;21)(q22;q22)*	0	5

*t(15;17)(q22;q21)*	0	4

Total	11	15

Distribution of AML patients without *MLL *rearrangements with MEIS1 deregulated according to karyotype information.

## Discussion

The presence of *MLL *rearrangements in acute leukemias results in a complex number of biological modifications that still remain largely unexplained. Armstrong et al. [9] proposed *MLL *rearrangement positive ALL as a distinct subgroup with a specific gene expression profile. However, this signature was related only to MLL with ALL phenotype. Here we show that MLL, from ALL and AML origin, shares a signature identified by a small set of genes suggesting a common genetic disregulation that could be at the base of the mixed lineage leukemia in both phenotypes.

Most *MLL *aberrations have been successfully characterized by cytogenetics, immunophenotyping, molecular biology and, recently, gene expression profiling.

In the present study, a total cohort of 218 patients was analyzed by microarray approach to identify common deregulated MLL targets shared by different leukemia phenotypes and to inspect *MLL *involvement in acute leukemias with different lineage origin.

We analyzed gene expression data of 140 (training set) and 78 (test set) pediatric samples carrying ALL (N = 106+62) and AML (N = 34+16), with (N = 24+13) and without (N = 116+65) *MLL *translocations. Supervised analyses on the training set identified two specific signatures according to lineage origin and MLL presence; the results have been subsequently validated on the test set. The genes *MEIS1*, *ZEB2*, *SRGAP2P1*, *TMEM30A*, *AK2*, *TMED2*, *HIPK3 *and *FAM62B *showed marked up-regulation in patients with *MLL *mutation, both in ALL and AML. GO analysis revealed a general regulatory role of these genes in developmental process, biological process, apoptosis and programmed cell death. Our results confirm the primary function of *MEIS1 *gene in regulating fundamental MLL leukemia-related and HOX genes processes. *MEIS1 *up-regulation was also observed in a subgroup of AML patients showing inv(16)-like signature in the absence of *MLL *mutations.

We further identified a phenotype-related signature that distinguishes lymphoblastic and myeloblastic acute leukemias. A set of 6 probes allowed for separation of ALL and AML with and without *MLL *mutation, including *PAX5*, *CD72*, *CSRP2*, *LOC100130458*, *EBF1 *and *TCL1A *genes. We corroborate the main role of *PAX5 *gene in orchestrating basic biological processes such as leukocyte and hematopoietic development [10,48-50]. Mullighan et al. [51] showed that the genes regulating B-cell development and differentiation are mutated in 40% of pediatric ALL and that *PAX5 *was the most frequent target of somatic mutation being altered in 31.7% of cases. Similarly, we showed that *PAX5*, *EBF1*, *CD72 *and *TCL1A *are tightly correlated in the capacity to distinguish lymphoblastic and myeloblastic characteristics also in the presence of *MLL *mutations illustrating the importance of B-cell receptor signaling pathway in this subset of leukemias. We stressed the role of *PAX5 *in repressing *PU.1 *(NF-kappaβ) regulated reporter gene as supported by their opposite expression in B lineage cells with *MLL *rearrangements [52].

Deregulation events of *CSRP2*, *TCL1A, CD72 *and *EBF *genes in acute pediatric leukaemia have been previously reported using gene expression profiling [43].

The role of *CSRP2 *gene in leukemogenesis still has to be investigated. Bach et al. [53] demonstrated that proteins like *CSRP2 *with LIM domains play important roles in embryo development and hematopoiesis. Moreover, Bégay-Muller et al. [54] showed that the LIM domain protein Lmo2 binds to AF6, a translocation partner of the *MLL *oncogene. CRP2 protein, encoded by *CSPR2 *gene, was shown to transactivate the proximal promoter region of IL-6 [55] whose receptors are expressed in pediatric ALL with the t(4;11)/AF4 translocation [56]. The overexpression of *CSRP2 *in *MLL *mutated samples distinguishing ALL and AML supports a novel role for *CSRP2 *gene in leukemia development.

## Conclusion

Our study demonstrates that a small subset of genes identifies leukemias with *MLL*-specific rearrangements and clearly separates acute leukemia samples according to lineage origin. The subset includes well-known genes and newly discovered markers that allow for characterization of ALL and AML subgroups, with and without *MLL *rearrangements.

## Abbreviations

The following abbreviations have been used throughout the manuscript. *ALL*: Acute Lymphoblastic Leukemia; *AML*: Acute Myeloblastic Leukemia; *MLL*: Mixed Lineage Leukemia; *SAM*: Significance Analysis of Microarray; *PAM*: Prediction Analysis of Microarray; *FAB*: French-American-British; *RMA*: Robust Multiple-array Average; *FDR*: False Discovery Rate; *GO*: Gene Ontology.

## Competing interests

The authors declare that they have no competing interests.

## Authors' contributions

AZ established study aim and design, performed gene expression analyses and wrote the manuscript, MCDO performed microarray experiments, GTK and GB supervised the study and writing of the manuscript. All authors read and approved the final manuscript.

## Pre-publication history

The pre-publication history for this paper can be accessed here:



## Supplementary Material

Additional file 1 **Patient information**. Supplementary information about the 140 patient cohort enrolled in the present study. Click here for file

Additional file 2 **SAM and PAM results**. SAM results for *MLL *translocation-specific (379 probes) and phenotype-specific (622 probes) signatures. In addition, PAM results (15 probes) are listed in a separated column. Probe set number and gene symbol have been provided for each analysis. Click here for file

Additional file 3 **Validation on Test Set**. Unsupervised hierarchical clustering of 78 pediatric leukaemia patients using the 15-probes predictor identified by SAM and PAM analyses. The dendrogram separates AML (red and blue labels) from ALL (green and orange) samples. Each group further divides into MLL-positive and MLL-negative samples. Click here for file

Additional file 4 **Gene Ontology results**. Gene ontology results for phenotype-specific (379 probes) and *MLL *translocation-specific (622 probes) signatures using DAVID web-based tool. Biological processes are ordered by FDR relevance; highlighted rows depict GO terms with FDR <10%. Click here for file

Additional file 5 **AML patients without MLL mutation and MEIS1 upregulated**. SAM results from the analysis of 26 AML patients without *MLL *translocation. Class comparison analysis was performed between AML/MLL- patients with MEIS1-upregulated and MEIS1-downregulated (see hierarchical clustering results for PAM analyses, Figure 3). Probes up-regulated in one group are considered down-regulated in the other, and vice versa. Click here for file
