# Supplementary figures and images for: MLL rearrangements in pediatric acute lymphoblastic and myeloblastic leukemias: MLL specific and lineage specific signatures
Source: BMC Med Genomics. 2009 Jun 23;2:36. doi: 10.1186/1755-8794-2-36 (PMC2709660; doi:10.1186/1755-8794-2-36)

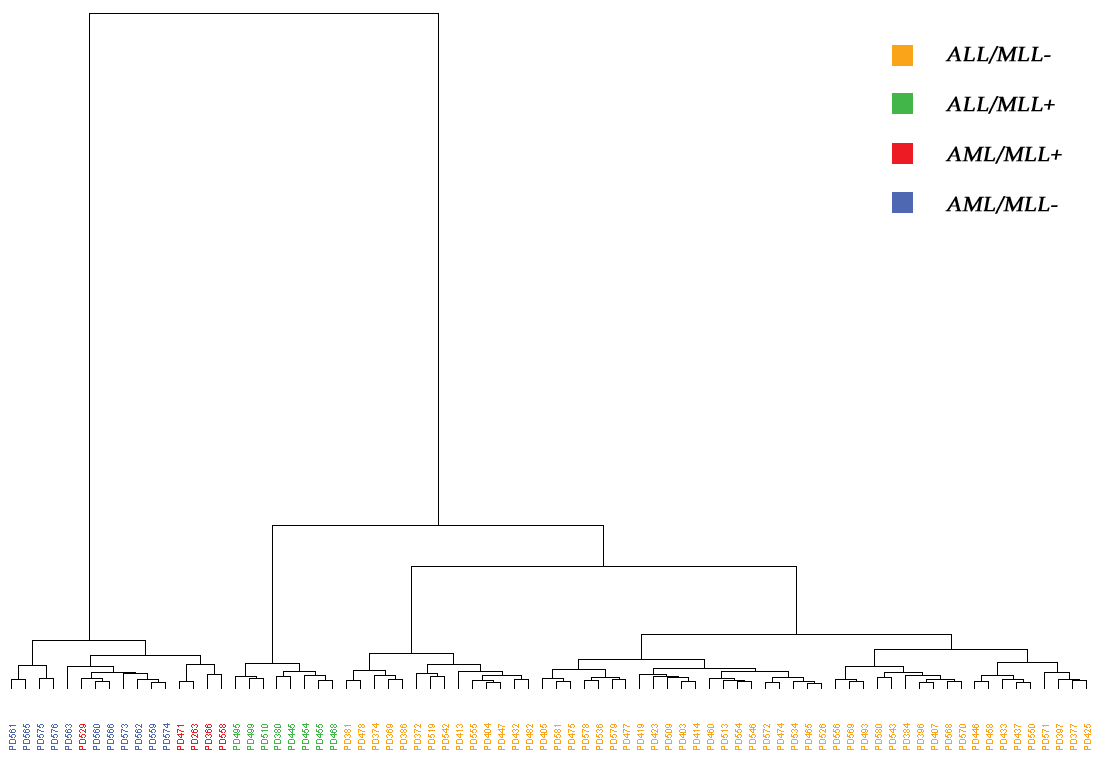

Supplement: Additional file 3 — Validation on Test Set. Unsupervised hierarchical clustering of 78 pediatric leukaemia patients using the 15-probes predictor identified by SAM and PAM analyses. The dendrogram separates AML (red and blue labels) from ALL (green and orange) samples. Each group further divides into MLL-positive and MLL-negative samples. [file 1755-8794-2-36-S3.png]
